# Flavivirus Persistence in Wildlife Populations

**DOI:** 10.3390/v13102099

**Published:** 2021-10-18

**Authors:** Maria Raisa Blahove, James Richard Carter

**Affiliations:** Department of Chemistry and Biochemistry, Georgia Southern University, P.O. Box 8064, Statesboro, GA 30460, USA; mb09692@georgiasouthern.edu

**Keywords:** viral persistence, flaviviruses, mosquito, tick, autophagy, interferon, wildlife, infection, arbovirus

## Abstract

A substantial number of humans are at risk for infection by vector-borne flaviviruses, resulting in considerable morbidity and mortality worldwide. These viruses also infect wildlife at a considerable rate, persistently cycling between ticks/mosquitoes and small mammals and reptiles and non-human primates and humans. Substantially increasing evidence of viral persistence in wildlife continues to be reported. In addition to in humans, viral persistence has been shown to establish in mammalian, reptile, arachnid, and mosquito systems, as well as insect cell lines. Although a considerable amount of research has centered on the potential roles of defective virus particles, autophagy and/or apoptosis-induced evasion of the immune response, and the precise mechanism of these features in flavivirus persistence have yet to be elucidated. In this review, we present findings that aid in understanding how vector-borne flavivirus persistence is established in wildlife. Research studies to be discussed include determining the critical roles universal flavivirus non-structural proteins played in flaviviral persistence, the advancement of animal models of viral persistence, and studying host factors that allow vector-borne flavivirus replication without destructive effects on infected cells. These findings underscore the viral–host relationships in wildlife animals and could be used to elucidate the underlying mechanisms responsible for the establishment of viral persistence in these animals.

## 1. Introduction

Understanding the mechanisms that underlie flavivirus persistence in wildlife and humans will be critical for the comprehension of persistent arboviral flavivirus infections. Arboviruses are arthropod-borne viruses. Arthropod is a word used to describe ticks and mosquitoes. Flavivirus infections constitute substantial human and non-human primate morbidity and mortality worldwide. Furthermore, the incidence is increasing, and infections are being appreciated in previously non-endemic locations, presumably due to the increasing rapidity of worldwide travel and deforestation practices [1,2,3]. Flaviviruses originating from tick and mosquito vectors comprise a crucial number of wildlife infections [4,5,6]. For example, some predominate tick-borne flaviviruses include Omsk hemorrhagic fever virus, Kyasanur forest disease virus, Alkhurma virus, Powassan virus (POWV), and deer tick virus (DTV) [7,8,9].

Mosquito-borne flaviviruses are best known through studies on Japanese encephalitis virus (JEV), yellow fever virus (YFV), West Nile virus (WNV), Zika virus (ZIKV), and dengue virus (DENV) serotypes 1–5. Infections with all these viruses can lead to severe disease symptomologies, prolonged debilitating neurological consequences, birth defects, hemorrhagic fever, and even death [10,11,12,13].

**Figure 1:**
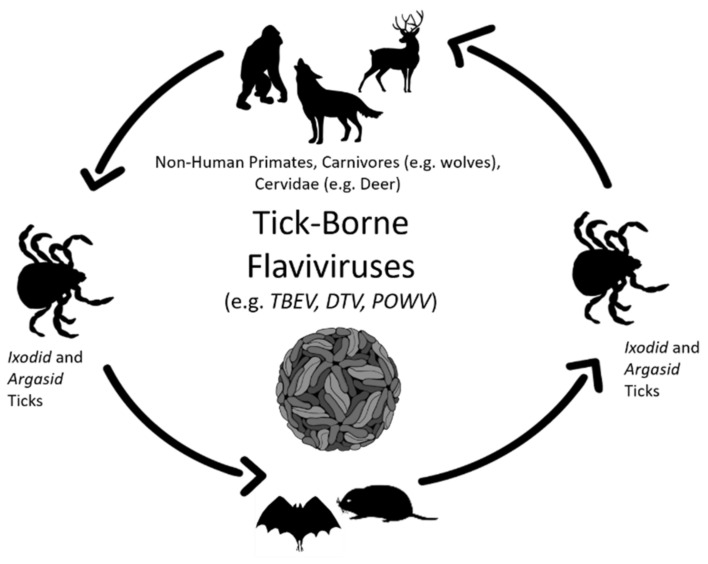
Schematic Representation of the Tick-borne Flavivirus Wildlife Transmission Cycle. Black arrows show the transmission cycle of tick-borne flaviviruses from *Ixodid* and *Argasis* ticks to predominate and intermediate hosts, as discussed in this review.

**Figure 2:**
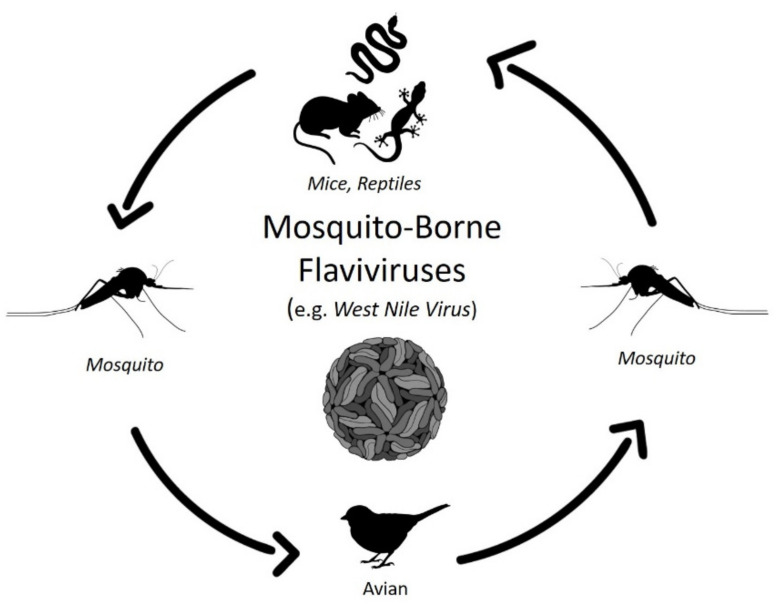
Schematic Representation of the Mosquito-Borne Flavivirus Transmission Cycle Involving Wildlife Hosts. Black arrows show the transmission cycle of mosquito-borne flaviviruses from mosquitoes (e.g., *Culex* sp.) to predominate and intermediate hosts, as discussed in this review. Unlike tick-borne infections, the transmission cycle for mosquito-borne viruses can differ greatly. For example, the transmission cycle for the West Nile virus (as shown above) differs from that of the dengue virus (DENV). DENV tends to circulate in two relatively distinct transmission cycles vectored by *Aedes* sp. mosquitoes. DENV infection of humans results in a sufficiently high viremia to support the infection of feeding mosquitoes. DENV may also replicate in a sylvatic cycle, which is more relevant to this review.

Viral persistence is a staple of arthropod-borne flavivirus pathogenesis. Mosquito-borne and tick-borne viruses are cycled between arthropod and vertebrate hosts (Figure 1 and Figure 2) and are predominately maintained without detrimental effects on host biology. In the natural world, tick-borne flaviviruses, such as POWV and TBEV, cycle between a range of hard-bodied (ixodid) ticks, soft-bodied (aramid) ticks, and small vertebrates, such as rodents, leporids, and some carnivores, such as wolves [13,14,15]. Similarly, mosquito-borne flaviviruses, such as Usutu virus (USUV), WNV and JEV, primarily cycle between mosquitoes and small mammals, birds, and reptiles (Figure 2; [16,17,18,19]). In addition, there is well-documented evidence that the persistence of mosquito-borne flaviviruses and tick-borne flaviviruses also occurs in cell culture [5,20,21,22,23,24].

**Figure 3:**
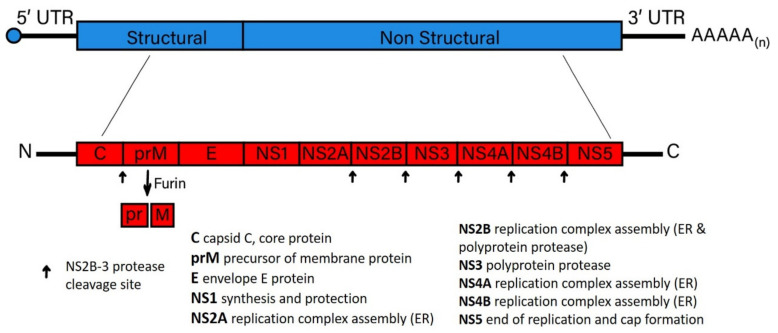
Schematic Diagram of the Vector-borne Flavivirus Genome. A representation of the approximately 11 kb flavivirus genome (in blue), capped and polyadenylated, and subsequent translation protein products (in red) are shown to illustrate the important flavivirus replication functions. These features and functions are consistent between tick-borne and mosquito-borne flaviviruses. Functions of the flavivirus genes as they pertain to the establishment of viral persistence in host cells are described in the text. UTR = untranslated region, AAAAA(n) = polyadenylation.

Flavivirus particles, regardless of whether tick-borne or mosquito-borne, are spherical and enveloped. These particles that contain RNA, 12 kb of positive-sense genome that encodes a single polyprotein that is cleaved into 10 proteins predominately by the NS2B-3 protease, except for the maturation digestion of prM into pr and a fully mature M. Once the virus particle is uncoated following infection, flavivirus RNA genomes are replicated. Following replication of the RNA genome, the polyprotein is translated, consisting of three structural proteins (capsid (C), precursor membrane/membrane (prM), and envelope (E)) and seven non-structural proteins, designated as: NS1, NS2A, NS2B, NS3, NS4A, NS4B, and NS5 [25,26,27] (Figure 3).

Due to the complex nature of flavivirus transmission between arthropods and vertebrates, it is likely that the process of flaviviral persistence is also very complex. Therefore, the justification for this review is to discuss the current situation in flavivirus persistence research, describe the host cell factors that play critical roles in the establishment of flavivirus persistence, useful yet unconventional detection approaches, and potential research endeavors into this crucial area of viral pathogenesis.

## 2. Infection and Persistence of Flaviviruses in Wildlife

Small mammals, marsupials, reptiles, and birds serve as the principal vertebrate hosts for vector-borne flaviviruses [28,29]. Interestingly, larger animals, such as deer, goats, and sheep, also serve as hosts, but more in an incidental capacity. It is worth noting that WNV in humans is thought to be associated with avian infection [30]. However, WNV migration from the Eastern United States to the West Coast by 2003 was undoubtedly due to infection and persistence of this virus in migratory birds, reptiles, and amphibians [31]. In addition to non-human primates, dengue infection has occurred in pigs, marsupials, bats, birds, horses, bovids, rodents, and canines [32,33]. There is potential enzootic transmission, but regular dengue virus spillback cannot be excluded. Except for bats, acute dengue infection among animals is still limited in evidence [34,35]. Similar to WNV, the newly emerging mosquito-borne Usutu virus (USUV) is also transmitted between mosquito arthropods and avian species [36]. USUV has also been shown to transmit between mosquitoes and canines, deer, and boars, as well as bats and rodents [37]. Moreover, for mosquito arboviruses, incredibly little is known about the nature of the vertebrate host [38]. Therefore, more work into the elucidation of host-species interactions is essential for the determination of flavivirus persistence in wildlife.

Small mammals, especially rodents, are the predominant vertebrate reservoirs and hosts for tick-borne flaviviruses [39,40]. In Europe, the most prevalent hosts are the bank vole and the yellow-necked mouse due to their ability to develop levels of viremia that are adequate for the infection of ticks that feed on them, thus allowing these animals to maintain tick-borne flaviviruses [41,42,43]. In Poland, captured bank voles, on average, displayed a TBEV seroprevalence rate of approximately 15% [44]. In North America, a number of wildlife animals, such as deer, squirrels, mice, and skunk, are significant reservoirs of DTV and POWV [45,46,47]. Serological studies detected a DTV prevalence in 6.2% of the red-backed voles found in Alaska and Siberia [48]. These findings show tick-borne flaviviruses display a broad range of distribution, seemingly regardless of geographical location.

DTV has previously shown the relatively high serological prevalence in Peromyscus truei and Peromyscus maniculatus mice [39,45,48], suggesting persistent infection may have occurred. Larger animals, such as deer, may preserve tick-borne flaviviruses at levels that permit persistence and tick-mediated transmission [7,49,50,51]. Even though deer are an essential source of nutrients for ticks, only low viral titers are achieved and are not directly involved in tick-borne flavivirus transmission. Nonetheless, deer play a critical role in flaviviral persistence because of their ability to support tick populations [39,52,53].

Understanding the importance of the sylvatic and urban cycles and their interplay is critical for the study of flavivirus persistence [54]. The sylvatic cycle exists between arthropod vectors and wildlife hosts, such as non-human primates or deer. The second cycle is the urban/domestic cycle, occurring between the vector and humans or even domestic pets. The urban cycle is highly influenced by ecological and social factors and is dependent on human behavior, leading to the transmission of vector-borne flaviviruses from the sylvatic cycle, where arthropod-borne flaviviruses flourish, into the urban cycle. This leads to increased human and wildlife infections. Evidence of this is the fact that vector-borne flaviviruses are becoming more prevalent in populations that were once non-endemic to these viral pathogens.

Research suggests that the principal reservoirs of mosquito-borne flaviviruses are non-human primates and avian species, often referred to as a “sylvatic cycle” [32,55]. Additional mammals generally serve as accidental hosts, but not always, as this still requires additional research [10]. When viremia is substantial, these animals possess the ability to pass these viruses on to female mosquitoes. For JEV, the major amplifying hosts are avian and swine, including wild hogs [56,57,58], which attain high levels of viremia.

In locations where swine are in short supply, avian species (e.g., herons and egrets) become the predominant amplifying hosts for JEV and the source of transmission for mosquito species that transmit this flavivirus to wildlife [59,60,61]. The persistence of JEV antibodies in pigeons has been detected for a period equivalent to 15 months [62]. However, more research is needed to determine if there is a correlation between antibody persistence and viral persistence. Furthermore, a critical role in JEV infection has been determined in a number of avian species. Nonetheless, evidence directly linking JEV avian infection to persistence in these species is in short supply [63].

As we alluded to earlier in this review, avian species also serve as exceptional amplification hosts for WNV. The predominant advantage here is that migratory birds can transport viruses great distances, due to the ability of birds to remain viremic for a number of days [64,65,66]. Furthermore, an incredibly high mortality rate (~100%) is associated with WNV infection in many bird species [67,68]. Almost surprisingly, some avian species can carry WNV for a longer time before developing antibodies or succumbing to disease symptoms [69]. For example, 37% of house sparrows that were infected with WNV tested positive for WNV RNA by RT-PCR [70]. Furthermore, 97% of house sparrows and 100% of finches tested seroconverted [70]. An interesting finding to note here is that research has also shown avian species that develop neutralizing WNV antibodies are protected from reinfection [71], suggesting absolute WNV clearance. However, this does not provide any correlation or contradiction between WNV infection and WNV-established viral persistence.

Blue-gray pigeons were the first avian species to show WNV persistence and have become a model system for the study of WNV persistence [70,72]. In blue-gray pigeons, WNV could be isolated for up to 100 days post-infection, and WNV antigens were detected in hepatocytes up to 180 days post-infection [73,74,75]. American robins are competent reservoirs of WNV and St. Louis encephalitis virus (SLEV) [76,77,78]. Therefore, one can presume sufficient evidence for persistent infection by WNV in some avian species has been obtained, but not for all bird species. However, many mechanistic details need to be elucidated before further claims can be made.

Similar to avian species, the antibodies of flaviviruses described above have been identified in turtles, snakes, and crocodiles from different regions throughout the world [79,80], and it is known that alligators and crocodiles can be infected with WNV. Conversely, no reports suggest that reptiles possess persistent mosquito-borne flavivirus infection, even though these animals have demonstrated facilitation of the transmission of these mosquito-borne viruses, especially WNV.

From the presented investigations, it is obvious that various animal species can possess persistent vector-borne flavivirus infection. However, additional research is essential for the further elucidation of the exact role persistent infections of vector-borne diseases play in the overall organization of viral maintenance. Lastly, the elucidation of host cell factors in the persistence of vector-borne flaviviruses is critical to the understanding of how these processes occur in wildlife and how, on a molecular level, host cell factors and their interaction with viral replication machinery (as an example) may enhance the persistence of flaviviruses in these members of the animal kingdom. It is also important to know considerably more about the mechanisms of viral persistence, as our knowledge base as a research body in this area is seriously lacking, as the current pandemic is demonstrating.

## 3. Mechanisms of Flavivirus Persistence

In this section, we will examine information linked to the instigation and preservation of flavivirus persistence. Since the literature regarding wildlife-related animal models is not available, our discussion in this section will be limited to studies involving in vivo cell culture and murine models, as they apply to the elucidation of the molecular mechanisms responsible for the manifestation of flavivirus persistence.

Infection of mammalian cell cultures with tick-borne flaviviruses results in the activation of a series of universal defense and antiviral systems, as they are precise factors designed to limit or restrict virus replication (see Table 1). As with a number of other viruses, some of these cellular factors include those involved in mitochondrial-activated signaling and the induction of inflammatory factors: interleukins, type I interferon (e.g., IFN-α/IFN-β), and type III interferon (e.g., IFN-κ) [81,82,83,84,85]. For instance, TRIM79α, an IFN-induced tripartite motif protein, restricts TBEV replication by inducing lysosome-dependent degradation of the flavivirus NS5 protein, an RNA-dependent RNA polymerase (RdRp) that is critical for virus replication [86,87]. This degradative event appears to be specific to tick-borne viruses, making the detection of TRIM79α overexpression a potential target for differentiating between mosquito-borne and tick-borne infections in wildlife populations.

Flavivirus truncated NS1 proteins have also shown a means to modify cell death. Sylvatic vector-borne flaviviruses, such as the TBEV Sofjin strain, YFV, DENV, Murray Valley encephalitis (MVE), and WNV, produce truncated NS1 proteins at a high multiplicity of infection [92]. The degradation of TBEV-NS1 proteins also restricts TBEV replication [115]. However, research in this area is very limited. In general, vector-borne flavivirus NS1 proteins differ in structural composition and cellular localization. In the cytoplasm of the cell, NS1 proteins are detected as a monomer [116]. In the ER and cellular membrane, NS1 proteins are detected as a homodimer [117]. Some protein molecules are secreted and form a hexamer outside the cell [118,119]. Diverse protein structures indicate diverse NS1 protein functions. This may even indicate diversity in NS1 protein processing, structure, and function among wildlife species.

Activation of cellular antiviral factors and systems often lead to cell death, which is thought to be primarily facilitated through apoptotic pathways [120], and programmed cell death induced by tick-borne flaviviruses have been described for a number of cell types, such as in hepatocytes, neurons, Kupffer cells, neuroblastoma cells, and epithelial cells [121]. Similar pathways have also been shown to result from mosquito-borne virus infection [122,123]. These observations indicate that tick-borne and mosquito-borne flaviviruses may initiate and establish viral persistence by similar mechanisms.

The inhibition or escape of a host’s antiviral response plays a critical role in viral persistence. IFN production is induced within hours of flavivirus infection [22,124]. However, because viral RNA replication complexes are enclosed in vesicle-like structures, IFN gene expression is delayed due to the inability of tors to recognize flavivirus RNA [125,126]. Furthermore, vector-borne flaviviruses can directly affect the expression of IFN signals by inhibiting the transcription of IFN genes and interferon-stimulated genes [127,128]. Moreover, humoral and cell-mediated immune responses can be inhibited by arboviral flaviviruses [129,130]. For example, the assessment of WNV infection of hamsters (a genetic cousin of voles) has suggested WNV neutralizing antibody production may decrease the spread of this virus to the host’s central nervous system [131,132,133]. Conversely, WNV variant-mediated antibody evasion could result in the evasion of T-cell recognition [133], but a precise role in viral persistence has yet to be determined.

Another feature of antibodies that needs to be considered is antibody-dependent enhancement (ADE). ADE occurs because during secondary flavivirus infection, cross-reactive yet non-neutralizing antibodies may lead to opsonization of this antibody on virus particles, resulting in an enhanced uptake by various immune cells, such as monocytes (in the case of DENV), resulting in amplified virus replication [130,134]. Elucidating potential implications of ADE in establishing flavivirus persistence may be crucial to understanding flavivirus evasion of T-cell responses.

The flavivirus E protein is believed to be accountable for the induction of the cytopathic effect [134,135], considering it promotes cell to cell fusion [136]. However, some flaviviral non-structural proteins may also play a role in the initiation of the cytopathic effect. For example, the IFN-independent cytopathic effect is facilitated by the DENV NS2A protein [137,138]. NS2B-NS3 protease precursor and NS3 protease have also been shown to induce apoptosis through interaction with caspase 8 [103,104,139]. Furthermore, of note, the intracellular expression of DENV NS1 alone is capable of initiating apoptosis [140,141]. These observations indicate a central role for apoptotic cascades in the induction of the cytopathic effect, leading to flavivirus persistence.

Elucidating the potential structure and functions of specific viral proteins expressed to block viral-induced cell death has been incomplete [142]. However, one key phenotype of note involves viral protein expression that limits cell death during acute infection, potentially enhancing the commencement of persistent infection. For example, flavivirus NS4A (Figure 3) was shown to promote the suppression of cell death through the induction of phosphatidylinositol 3-kinase (PI3K)-dependent autophagy [107]. JEV infection activates PI3K, which is thought to afford protection from the initial stages of cell death [143,144]. WNV pathogenesis is also dependent on the PI3K pathway. The treatment of cultured cells with 3-methyladenine (3-MA), a PI3K inhibitor, enhanced WNV in a dose-dependent manner [145], showing the impact autophagy may have on mosquito-borne flavivirus persistence in wildlife. However, more research is needed to determine if this hypothesis is valid and whether it occurs in a species-dependent manner. Tick-borne flaviviruses are also dependent on the PI3K pathway to maintain persistent infection. For example, the PI3K-AKT pathway has been implicated in TBFV persistence through assessing AKT suppression mediated by Langat virus (LGTV) infection [50]. Therefore, it can be surmised that the suppression of PI3K-AKT is essential for the maintenance of flavivirus persistence in wildlife populations.

Substantial attention has been focused on defective interfering (DI) virus particles, which have been shown to inhibit replication of the wild-type flaviviruses [146,147,148,149]. The DI virus particles contain truncated viral genomes that can be transcribed to a very high copy number and compete with wild-type viral genomes during encapsulation and assembly stages of virus replication [150]. Furthermore, these defective particles also compete with wild-type flaviviruses in infected cells. However, the potential roles of vector-borne flaviviruses DI particles in persistence are not transparent.

For TBEV, the C protein is reported to tolerate internal deletions that favor attenuation and immunogenicity [74,151]. Another tick-borne flavivirus, LGTV, also produces DI particles [152]. In this study, after 15 passages of LGTV, DIs constituted approximately 35% of the total LGTV population. Furthermore, at this point, the predominant DI population included a genome in which nucleotides 1058 to 2881 had been ablated. This defective vRNA genome encoded an intact polyprotein possessing a truncated fusion protein containing 28 N-terminal residues of E and 134 C-terminal residues of NS1. The role of this DI in establishing the persistence of this virus and potentially other tick-borne flaviviruses still needs to be elucidated.

Mosquito-borne flaviviruses also produce DI particles as part of their replication cycle, with WNV and DENV being the most studied to date. Fragments of dengue virus RNA containing only the key regulatory elements at the 3′ and 5′ ends of the genome were detected in the sera of patients infected with any of the four DENV serotypes [149]. Identical RNA fragments were detected in the supernatant from cultures of *Aedes* mosquito cells that were infected by the addition of sera from dengue patients. This result suggests sub-genomic RNAs might be transmitted between human and mosquito hosts in defective interfering (DI) viral particles, potentially giving insight into how mosquito-borne viruses remain persistent in host cells.

Previously in this review, we described the potential roles various structural forms of flavivirus NS1 play in the maintenance of flavivirus persistence in wildlife populations. Additionally, the expression of the truncated form of NS1 has also been shown to possess a link to DI virus particle production and persistent infection. For example, a truncated Murray Valley encephalitis virus (MVE) NS1 protein was detected during persistent MVE infection of mammalian cells. However, this form of NS1 was not detected during acute infection [74,153]. Moreover, truncated NS1 in the MVE virus appears to be the result of DI vRNA present in the infected cells. What is significant about these vRNA transcripts is they possess a substantial internal deletion, resulting in a significant reduction of the wild-type MVE titer [154]. This result gives additional insight into the role of truncated NS1 and DI in wildlife populations. However, an immediate role for MVE DI particles in preserving persistent infection was established.

The analysis of tick-borne encephalitis virus Far Eastern subtype (Sofjin virus) associated a 39-kDa truncated form of NS1 with acutely and persistently infected cells [93]. Although DI particles and truncated NS1 may be commonly observed in persistent flavivirus infections, it is not at all transparent that they are autonomously adequate for the establishment and maintenance of persistent infections in vitro or in vivo.

For WNV, DI particles marginally influence pathogenesis in mice while inhibiting transmission in mosquitoes and exotic birds [155]. Studies regarding DI-induced WNV persistence in other animal hosts, such as additional avian species and reptiles, would be quite significant in the elucidation of WNV persistence in wildlife as these hosts are in the same sylvatic cycle.

The surveillance of mice and hamsters infected with mosquito-borne flaviviruses obtained from the urine of other infected animals do not show symptoms of chronic infection and become persistently infected, suggesting virus attenuation [156]. Research has suggested this attenuation may be the result of several mutations in flavivirus genes: C, E, NS1, NS2A, NS2B, and NS5, and may be associated with WNV, JEV, and DENV persistence in wildlife species [157,158,159]. Therefore, the question remains: what mutations in flavivirus genes are responsible for the establishment of persistence by vector-borne flaviviruses? Furthermore, are these mutations wildlife species specific?

Mechanisms that do not directly necessitate viral protein products, i.e., activate host antiviral mechanisms instead, may also play a critical role in viral persistence. JEV express noncoding short RNAs that suppress host cells dsRNA sensing mechanisms through the inhibition of IRF3 phosphorylation [124,160,161]. WNV delays pathogen recognition receptor activity by activating IRF3 in a RIG-I-dependent manner [162], although the elucidation of the mechanisms surrounding this mode of escape and evasion remains elusive.

Membrane-bound vesicles that encapsulate the dsRNA of TBEV-expressing dsRNA have been discovered [163]. These results have been further investigated in cultured cells. However, the elucidation of the role of interferon-regulated proteins in TBEV persistence has been chiefly investigated in human cell lines and may not reflect the same in cultured cells of other species. As the suppression of mechanistic alterations to flavivirus recognition pathways are instituted for the establishment of flaviviral persistence.

Several host genes that may play roles in the development of flavivirus persistence in wildlife animal hosts have been suggested. These include proto-oncogenes, such as Bcl-2, and murine-derived alleles, such as Flvrm, Flvr, and Flvs. Over-stimulation of Bcl-2 has been reported to block apoptosis and promote JEV persistence in cultured cells [103,164]. This suggests that the control of apoptosis is likely to play a critical role in the establishment of viral persistence. Moreover, Flvrm, an oligoadenylate synthetase gene that potentially promotes resistance to flavivirus infection, appears to be present in some laboratory mice strains [165,166]. Mice that are susceptible to flavivirus infection tend to carry Flvs [167,168]. Flvr has been identified in wild mice and could, in part, aid in the elucidation of flavivirus persistence [169]. The expression of additional genes may play a critical role in the varied susceptibility observed in different mouse strains to flavivirus infection. Furthermore, the isolation and characterization of these genes from wildlife species could give further insight into the establishment of flavivirus persistence in the wild.

The establishment of flavivirus persistence in wildlife hosts may also be exacerbated by forms of immunosuppression. Very little is known of arbovirus-mediated immuno-suppression in wildlife species, so we must limit our discussions to human infections and the few bodies of research involving murine models. Arbovirus infection may establish persistence in organ transplant patients due to the use of immunosuppressive therapies [170]. In B6 mice, transient immunosuppression with cyclophosphamide leads to WNV re-emergence [171], suggesting aspects of host immune response serve to restrict replication of WNV during persistence. However, determining these immune functions, in this case, is further complicated by the observation that WNV can persist for as long as 16 months, even in the presence of over-stimulated humoral immune response in C57BL/6 mice [74,171]. Moreover, WNV persistence was reported in the brains of CD8+ T cell-deficient rodents [172,173], but antibody response was not affected by CD8+ T cell deficiency. Therefore, CD8+ T cell response only describes a small piece of the role immunosuppression may play in flavivirus persistence.

Precise immune system factors that could facilitate or regulate flavivirus infection persistence remain to be elucidated. The infection of host arthropod cells by arthropod-borne flaviviruses has not received the same level of attention. This may be due, at least in part, to acute infection not being accompanied by the same level of cytopathic response as observed in mammalian cells. Regarding tick-borne flaviviruses, very little is known about how these flaviviruses persist in the ticks but possibly evolved mechanisms of modifying and/or eluding tick immune responses [174]. Detailed studies of persistent tick-borne flaviviruses infection in arthropod cell lines using biochemical and molecular biology-based techniques and methods would yield useful and interesting data. The mechanisms surrounding the establishment of mosquito-borne flavivirus persistence in mosquitoes also remain an ideal topic of scientific investigation. A mosquito-borne flavivirus, St. Louis encephalitis virus, persists in *Culex pipiens’* midguts for several hours before infecting cells that are responsible for maintaining the midgut [175,176,177]. This brings to light interesting questions regarding the role of mosquito midgut cells in the establishment of flavivirus persistence in mosquitoes.

Versatile roles for miRNAs (MicroRNAs) in multiple biological processes are continually elucidated. However, little is known about the effects miRNA expression may have on flavivirus-persistent infection. A miRNA array analysis of Japanese encephalitis virus (JEV)-infected cells was performed to search for persistent infection-associated miRNAs in comparison to acute infection [178]. Among all differentially expressed miRNAs, the miR-125b-5p is most significantly increased [178].

Clearly, research into the mechanisms surrounding flavivirus persistence in vectors, such as mosquitoes and ticks, has been inadequate in the volume performed and is essential to complete the picture of how flavivirus persistence may occur. It is apparent that flavivirus and host cell-derived molecules play critical roles in the commencement and conservation of persistent infection in individual cells, and the host organism, as a whole. Lastly, environmental and ecological factors, such as climate change, may be driving forces in vector-borne flavivirus persistence and should certainly be investigated thoroughly.

One way the mechanisms described here is through co-infection of host cells with two or multiple vector-borne flaviviruses. Flavivirus co-infections in wild animals are examined using classical approaches that are based on sample collection, the method used to detect a particular infectious agent, and the results analysis method. Successful sample collection is generally achieved through cross-sectional or longitudinal studies [179]. Cross-sectional studies provide data on the co-occurrence of infectious agents at the time of sample acquisition, whereas longitudinal studies afford more detailed evidence regarding infection dynamics in individual hosts and groups over time. Nevertheless, the analysis of co-infections in wildlife are limited by biological sample obtained (e.g., urine, saliva, feces, and blood), and these samples are analyzed with targeted assays (e.g., PCR and serology) [180,181]. The advancement of next-generation sequencing, bioinformatics, and metagenomics provides methods to concomitantly describe many pathogens without prior knowledge [182,183]. Statistics of these assays, as all forms of experimentation, should be considered. Statistical tests, such as the chi-squared test, allow the rapid scrutinization of co-infection, but often with limited speculation concerning flavivirus interactions and their repercussions [179,184]. Many additional mathematical models, statistical tests, and ecological theories have been developed to better infer interactions, although approaches vary depending on study designs and infectious agents [185,186,187]. Field studies can also be associated with experimental approaches, such as captive studies [188].

The detection of TBEV antibodies occurred often as analyzed in all plasma samples from goats in a specific virus neutralization test (VNT) [189]. Virus neutralization assays measure neutralizing antibodies in serum and plasma, and the plaque reduction neutralization test (PRNT) is considered the gold standard for measuring levels of these antibodies for many viral diseases. We have developed procedures for the standard PRNT, microneutralization assay (MNA), and pseudotyped virus neutralization assay (PNA) for severe acute respiratory syndrome coronavirus 2. The MNA offers advantages over the PRNT by reducing assay time, allowing increased throughput, and reducing operator workload while remaining dependent upon the use of the wild-type virus [190]. The TCID50 (Median Tissue Culture Infectious Dose) assay is one method used to verify the viral titer of a testing virus. Host tissue cells are cultured on a well plate titer, and then varying dilutions of the testing viral fluid are added to the wells.

Increased intensity of disease severity may arise if two or even multiple arboviruses synergize and supplement each other’s replication in vivo. DENV, ZIKV, and the alphavirus family member Chikungunya viruses (CHIKV), are tropic for many of the same cells, resulting in similar disease symptoms, and disrupt the immune responses via similar mechanisms [191,192,193]. For example, the chikungunya virus inhibits the nuclear transport of STAT1 and the DENV virus blocks STAT2 phosphorylation [192]. Through these actions, the replication of both CHIKV and DENV co-infection may enhance replication of each virus in concert. Another example involves WNV and USUV co-infection. High titers of neutralizing antibodies against both WNV and USUV were previously detected following co-infection [194]. The molecular interplay between WNV and USUV co-infection and its role in viral persistence has yet to be elucidated. The potential persistence in flavivirus infection may be independent of the co-infection of WNV and USUV since these two viruses have shown differing sensitivity to interferon signaling [195]. A third example of co-infection potentially leading to enhanced flaviviral persistence involves triple co-infection of mosquito cells with DENV, densovirus (DNV), and JEV. Successive challenges of mosquito cell cultures followed by serial passages gave rise to stabilized cultures with all cells analyzed co-infected with DENV and DNV. The addition of JEV resulted in stable and persistent triple-virus co-infections of all three mosquito-borne viruses [196]. Additional outcomes of mosquito-borne virus co-infections may result. For instance, infection with multiple arboviruses may trigger a robust nonpathogenic antiviral state, reducing overall viremia and disease severity [197], many of which are the result of the presence of defective interfering (DI) particles [149,198]. Competitive simultaneous infection between multiple infecting arboviruses may result in identical transmission rates compared to a single infection with the most replication-competent virus [199,200]. Therefore, it is possible that if one virus replicates faster upon initial infection, it can infect cells first and use up cellular resources for its own replication, resulting in increased susceptibility of the same cells to the other viruses that are in the co-infection group. Knowledge regarding co-infection studies with tick-borne flaviviruses has centered on co-infection of tick-borne viruses, such as Powassan virus or tick-borne encephalitis virus, with non-viral pathogens, such as the bacterium Borrelia burgdorferi, the causative agent of Lyme disease [201]. Therefore, it is obvious that a new research field involving co-viral infection in ticks is long overdue.

## 4. Detection

Previous sections focused on the transmission through wildlife; however, the detection of vector-borne flavivirus infections should also be addressed. Vector-borne viruses that cross between multiple hosts mediated by arthropod vectors, such as ticks and mosquitos, utilize parasitism relationships between the host and these vectors, making the need for close monitoring ever more essential [202]. Careful monitoring of vector-borne flaviviruses following zoonoses can be useful in determining mechanisms essential in establishing flavivirus persistence in host species populations.

Extraction of flavivirus RNA in mosquito populations suspected of harboring the virus in question is a critical step in establishing mechanisms responsible for mosquito-borne flavivirus persistence. Knowing where a flavivirus prefers to replicate may aid in simplifying the study of flaviviral persistence. A mosquito fat body is a diffused organ and is similar in function to a mammalian liver and is regulated for metabolism and nutrient storage in addition to antimicrobial peptides production and secretion, as well as other immune-based molecules [203,204]. In the mosquito hemocoel (i.e., mosquito body cavity), flaviviruses replicate in the abdominal and thoracic fat body prior to dissemination to the salivary glands and other tissues [205]. For example, WNV replicates *in Culex pipiens quinquefasciatus*, principally in fat bodies [206], but DENV replication in fat bodies has yet to be linked with DENV infectivity in *Aedes albopictus* and *Ae. aegypti* mosquitoes [207,208]. However, additional research indicates DENV replication in the fat body cells of *A. albopictus* alters the expression of cytoskeleton proteins Actin and alpha Tubulin [209] and downregulates Toll pathway-related gene transcription in Ae. aegypti mosquitoes [210,211].

West Nile virus (WNV) RNA can be isolated directly from the cerebrospinal fluid (CSF) of infected hosts. One effective approach involved subjecting CSF to unbiased metagenomic deep sequencing that was boosted with the use of a Cas9-based technique [212]. Histopathological and ultrastructural analyses techniques can be used to reveal the presence of flaviviruses in target organs, such as liver, lung and heart, in human and wildlife samples [10,200,213,214]. Post-mortem samples tend to show tissue abnormalities, such as necrosis and apoptotic cell death of tissue, to show inflammation that is characteristic of disease outcomes resulting from flavivirus infections, such as those resulting from severe dengue [213].

Target organs and viremia of tick-borne flavivirus RNA are also essential for the determination of potential flavivirus persistence. In bank voles, acute infection, infection kinetics, and viral persistence of TBEV were assessed using the three known TBEV subtypes: Siberian (TBEV-Sib), European (TBEV-Eur), and Far Eastern (TBEV-FE) [215,216]. TBEV RNA was initially (at approximately 4dpi) detected in the brains more often than in the kidneys or serum. At this stage, lungs and spleens did not contain TBEV RNA any less frequently than the brain, but as infection progressed, the replication of viral RNA dropped aggressively in these organs in comparison to the brain [40,215,216].

Early detection of vector-borne flavivirus infection is largely focused on the collection of viral RNA for molecular detection and genotyping. Mosquito and tick vectors are collected and processed for reverse transcription-polymerase chain reaction (RT-PCR) to detect viral RNA. RT-PCR utilizes heat cycles to denature viral RNA, an annealing primer to target and extend a specific sequence [217]. Genotyping of the RT-PCR samples utilizes a form of quantitative PCR (qPCR) that includes the addition of a fluorescent marker to identify key fragments in the RNA sequence [217]. In short, the PCR-based detection of viral RNA can be useful in the early detection of flavivirus infections starting in common viral vectors and wildlife containing the tick-borne or mosquito-borne flavivirus in question. The results obtained can be used to determine RNA copy/titer as it relates to the persistence of the flavivirus being analyzed.

Once the flavivirus is transmitted from the vector to hosts, the need for monitoring switches from vector to host, realistically, in most areas of the world, the focus on the detection of flaviviruses in vectors occurs only after an outbreak of infections following the onset of symptoms known to have wildlife vectors [217,218,219]. The lack of entomological surveillance in wildlife populations allows for flaviviruses to go undetected, eventually resulting in established persistence of various wildlife-based diseases and eventual transmission to human hosts.

In medical and research facilities around the globe, the conformation of viral activity is typically detected through antibody tests or RT-PCR [220]. Antibodies found in the bloodstream, such as immunoglobulins G and M (IgG and IgM), can be quantified through enzyme-linked immunosorbent assays (ELISA) and specifically the neutralizing antibodies that correspond to the infection that can be confirmed [221]. Typically, IgG and IgM are present in serum samples. However, IgM antibodies are short-term reactions to infections and last up to 10 months from initial infection, while IgG antibodies are considered to last a lifetime [220]. It should be noted that there is a period shortly after initial infection that a host has yet to begin the production of viral antibodies, and thus the possibility of a negative antibody test is not always indicative of a negative diagnosis. Therefore, antibody tests should be confirmed with RT-PCR detection methods when applicable [222].

High-throughput multiplex detection of many viruses, including flaviviruses, has been performed using DNA microarray [223,224,225,226,227]. Nevertheless, this method possesses many limitations, including: expensive, labor-intensive, and very time-consuming. Most notably, the hybridization process may take hours to days to complete. Then, nonspecific hybridization between sample components and immobilized probes compromise assay sensitivity. Additionally, the designing of probes requires precise information of the genetic makeup of the virus(es) of interest. The assay detects only those viruses that have target probes on the array [228,229,230].

The amplification of RNA targets is achieved through RT-PCR. In this technique, reverse transcriptase (RT) is used to alter viral RNA targets into complementary DNA (cDNA). The resulting cDNA is then amplified by conventional PCR. RT-PCR has been used for the diagnosis of human infection by RNA viruses. In the detection of viral infection, conventional RT-PCR has demonstrated sensitivity in the range of 73% to 100%, with a specificity of 99% to 100% [29,57,58]. These data indicate that RT-PCR is an excellent technique for the diagnosis of human infection of RNA viruses. Nowadays, however, the method is costly and time-consuming [14]. A very recent development involves a method that couples RT-qPCR and microarray technologies. A novel assay that combines SYBR Green-based RT-qPCR with a low-density DNA microarray has been developed for the detection of all known flaviviruses [231]. This assay has been used to detect flaviviruses present in laboratory and field samples (i.e., wildlife samples) and in international external quality assessment of nucleic acid amplification tests (NAAT) [231,232].

The presence of flavivirus RNAs and host species DNA/RNA can be detected in blood-fed female mosquitoes [224] as well as in tick arthropods [233]. Amazingly, blood obtained from the midgut of female mosquitoes can be analyzed to determine which species of wildlife the female mosquito in question had recently fed on, which flaviviral pathogen had been transmitted, or which species of mosquito is the culprit in the transmission event [234,235,236]. Successful detection can be achieved through ELISA assays to detect flaviviruses with antibodies to a particular antigenic site on the E protein or by multiplex PCR with primers designed to target suspected host DNA or viral RNA [236,237]. What is intriguing is the diversity of targets one can choose if the detection by PCR-based approaches is desired. For instance, one can choose to target mammalian or mosquito species-specific DNA encoding cytochrome B [224,233], microsatellite loci DNA [233,238,239,240,241], or species-specific cis-regulatory elements may be chosen for analysis, such as Aves class-specific cis-regulatory elements in the case of avian species [242,243]. In short, using molecular techniques is a powerful tool in the detection and characterization of host and mosquito/tick species that is transmitting the vector-borne flavivirus in question.

Advances in viral antibody sensitivity in clinical settings have occurred exponentially in response to recent advances in nanotechnology. The ELISAs mentioned previously are the most frequently utilized methods that detect and quantify critical concentrations of flavivirus-specific antibodies. However, current methods require specialized conditions for storage and use. Marker enzymes requiring low temperatures and/or preservation through solvents that serve as suitable alternatives are on the rise [222].

A promising alternative rooted in low-cost, high sensitivity, and easy miniaturization is the use of nanoparticles. Bioconjugation of stable colloids of metal nanoparticles, such as silver (Ag) or gold (Au) nanoparticles, to label immunoreagents, is one such approach [222,244]. The bioconjugates of Ag nanoparticles, for example, are prepared alongside flavivirus-specific antibodies, and it is the silver that acts as a direct signaling marker through silver chloride reduction on a gold-carbon composite electrode (GCCE), which is easily monitored through cathodic linear sweep voltammetry or CLSV [222,244]. This method increases flavivirus detection sensitivity and selectivity without requiring specialized storage conditions. However, additional engineering is needed to increase the compatibility of detection apparatus to allow for virus detection in the wild.

The issues surrounding detection apparatus portability may be solved by altering nanoparticle bioconjugates. Nanoparticles are also utilized in various rapid detection methods, often marketed as a rapid, cost effective way of actively tracking viral infections. Scientists have been successful in coupling DNAzyme activation with salt-induced aggregation of metal nanoparticles, such as gold-based particles, in a manner that allows for the recognition of cyclization sequences specific to key viruses, such as the dengue virus [245].

## 5. Potential Areas of Future Research Endeavors

Every vector-borne virus studied to date that causes disease in humans antagonizes interferon type 1 (IFN-1) signaling by disrupting the JAK-STAT signaling pathway [246], suggesting suppression of this pathway will interrupt vector-borne flavivirus persistence. Thus far, we discussed the significant literature with relevance to flavivirus persistence in wildlife populations. The understanding of how flaviviruses persist in wildlife species could aid in the advancement of therapeutic treatments that could suppress the potential for transmission to human populations. Unfortunately, applicable animal models are limited. Therefore, we suggest the development of animal models based directly on flavivirus infections that have been detected in wildlife species. For example, investigations with guinea pigs and ferrets for immunologic investigation with JEV have been successfully performed [247], suggesting their use as animal models for the study of flavivirus persistence. In fact, for a significant amount of time, ferrets have served as a powerful model system for the study of influenza pathogenesis due to this animal’s ability to exhibit symptoms consistent with influenza virus infection. Therefore, it stands to reason that ferrets may make for an excellent animal model for the study of vector-borne flaviviruses in wildlife.

Despite the amount of research that has been performed to date in flavivirus persistence, sustained research is essential to understanding the pathogenesis and persistence of these significant wildlife-based pathogens. For example, the defined roles of specific viral proteins and cell-based molecules and their interactions during the formation and maintenance of persistent infection are very inadequate. Furthermore, for flaviviruses to persist in infected cells, whether in vitro or in vivo, precise host defenses need to be eluded or suppressed. In short, several basic questions need to be addressed: are cellular factors responsible for the establishment of flavivirus persistence or are viral proteins the culprits? Which factors, specifically? Or are both viral and host proteins responsible but at different instances of the infection and persistence process?

A potential role NS1 plays in establishing viral persistence has been described earlier in this review as it applies to the ability of flaviviruses to influence defective interfering virus particle production. NS1 may also play another potentially vital role in establishing viral persistence: interferon (INF)—type 1 antagonism. Interestingly, flavivirus NS1 proteins have been shown to possess several roles in pathogenesis; from vascular leakage in DENV infection to reducing deactivation of extracellular viruses by binding to the C4 (e.g., TEBV NS11) [71], to bind a component of the complement system to inhibit INF activity. However, in other virus families, such as orthomyxoviruses (e.g., influenza A) and paramyxoviruses (e.g., respiratory syncytial virus), their respective NS1 proteins have demonstrated a role in INF type 1 antagonism [248,249,250,251]. Therefore, it stands to reason that NS1 proteins originating from flaviviruses that circulate among wildlife populations (e.g., DENV and WNV) may play a significant role in INF antagonism. Moreover, it bears mentioning that Zika virus NS1 proteins from PVABC-59 and Dakar-41525 strains suppress INF-β induction by 40% and 30%, respectively [252]. Zika virus has yet to show significant presence in wildlife, as opposed to other vector-borne flaviviruses in this review and is thus beyond the scope of this review to mention any further.

The flavivirus non-structural protein 5 (NS5) is an N-terminal methyltransferase (MTase) domain tasked with creating the cap1 structure at the 5′ end of nascently synthesized vRNA genomes, which promotes translation [253] and suppresses viral RNA detection by host immune responses [254]. Flavivirus non-structural protein 5 (NS5) has shown to be the most potent IFN antagonist for all illness-causing flaviviruses to target diverse steps of the type I IFN signaling pathway [128], suggesting a role for NS5 in the establishment of flavivirus-mediated infection persistence. The role of tick-borne virus NS5 proteins in the antagonism of interferons during infection has been established [255]. However, its IFN antagonism in the framework of vector-borne flaviviruses persistence has not been completely investigated. However, significant progress has been made in the role NS5 plays in altering INF signaling, which can lead to enhanced vector-borne flavivirus persistence.

Interestingly, STAT 2 is a favorite target of flavivirus NS5 proteins for the modulation of IFN signaling cascades to establish flavivirus persistence. ZIKV NS5 induces STAT2 degradation. STAT2 is essential for the development of transcription complexes involved in type I and III IFN signaling. These transcription complexes promote the intracellular accumulation of STAT-containing complexes leading to STAT1-STAT1 dimer formation, resulting in augmented IFN-γ-induced gene expression [128]. DENV NS5 binds to STAT2, inhibiting its phosphorylation and resulting in the reduced transcription of Interferon-Stimulating Genes (ISG). The mechanism of this NS5-mediated inhibition has yet to be elucidated, but IFN-induced suppression has been mapped to the RNA-dependent RNA polymerase binding domain [111]. Even though DENV NS5 does not directly interact with STAT1, IFN-α-mediated STAT1 phosphorylation is reduced [111], suggesting the involvement of additional host cell factors. YFV NS5 also targets STAT2 as part of its IFN-I suppression mechanism [128]. This is achieved through YFV NS5 binding to STAT2 but is solely dependent on host cell provocation with type I or III IFNs [112]. This stimulation event encourages several intracellular events required for NS5-STAT2 association with STAT2 [255]. Interestingly, WNV displays a similar mode of action against INF type 1, through activity against the JAK-STAT pathway as has been described for DENV NS5, suggesting similar pathways are utilized to achieve viral persistence for both DENV and WNV.

The mosquito-borne flaviviruses NS4B protein, which inhibits JAK/STAT-mediated IFN-α/β induction [256,257], is also of note. Moreover, mutations in NS2A of the WNV strain Kunjin virus result in amplified intracellular IFN concentrations, suggesting NS2A-mediated antagonism of IFN antagonistic is a possibility [193,258]. IκB kinase ε (IKKε) and TANK-binding kinase-1 (TBK-1) are two predominate kinases responsible for IRF3 activation and are also targeted by flaviviruses. The DENV2 protease, NS2B3, interacts with IKKε, preventing IRF3 phosphorylation and subsequent nuclear translocation [259]. Furthermore, TBK-1 can be inhibited by the flavivirus non-structural proteins NS2A and NS4B, as determined in all DENV strains tested, by NS4A of solely DENV1, and by NS4B of WNV [260]. Expression of these non-structural proteins in infected cells has been shown to inhibit TBK-1 autophosphorylation and subsequently IRF3 phosphorylation and type I IFN transcription [124,260]. Altogether, these studies demonstrate that mosquito-borne flaviviruses, such as DENV and WNV, are invested in antagonizing essential cellular proteins that produce type I IFN. Nevertheless, more research is required to fully understand the mechanisms involved and how these mechanisms facilitate the establishment of flavivirus infection persistence in wildlife hosts. The elucidation of pathways critical for switching or molecular/genetic alterations that may be critical for the transmission of flaviviruses from arthropod vector to wildlife host is also interesting.

Precise identification of host immune responses that are eluded and/or controlled in specific wildlife species is critical. This may include the identification of pro-apoptotic proteins involved in the establishment of flavivirus persistence. Understanding these mechanisms in arthropod vectors that transmit these viruses are also critical. Bcl-2 overexpression in BHK and CHO cells resulted in the inhibition of apoptosis and JEV persistence, even though a direct link to viral effect on Bcl-2 activity was not determined [164]. The results revealed the overexpression of Bcl-2 in BHK-21 cells, although not inhibiting virus yields, delayed the process of DENV-induced apoptosis, thereby permitting surviving cells to become persistently infected [261]. It would be advantageous to understand which and how vector-borne flavivirus proteins interact with the Bcl-2 pathway in mosquitoes and ticks, as well as wildlife animals. Overexpression of tBax, a powerful inducer of cellular apoptosis, in mosquito C6/36 cells resulted in the inhibition of DENV infection [262,263]. Undeniably, other host cell pathways could be involved in the establishment or blockage of flavivirus persistence.

Intimate connections of persistent flavivirus infection in the tick and mosquito vectors also need to be elucidated. The biology surrounding flavivirus persistence in ticks and wildlife species is completely divergent. Regarding ixodid tick vectors, identifying genomic, proteomic targets that may play a role in viral persistence is imperative to elucidation. When known, these vector mechanisms might be useful in efforts to control natural flavivirus persistence cycles in ticks. For instance, these mechanisms could involve specific mutations that may render the virus less detectable by immune responses in infected cells.

An important question for transmission and flaviviral persistence mechanism dynamics is why arthropod vectors are not killed by vector-borne virus infection. Analysis of vector-borne flavivirus-mediated IFN antagonism has been predominately performed to date in the context of human disease. Even studies in animal systems are typically performed in humanized mice [264,265]. This is not conducive to the assessment of arthropod-borne persistence in wildlife species. Therefore, a more conscious effort should be made for the study of vector-borne flaviviruses in the wildlife species they cause disease in. This should include more mammalian-based (non-human) cell culture research and rigorous fluid and tissue sampling programs geared towards the specific collection of samples from flaviviruses detected and analyzed for viral persistence.

Taken together, these findings suggest the INF-induced JAK-STAT pathway is the predominant player in the establishment of vector-borne flavivirus persistence.

Lastly, establishing animal models of vector-borne flaviviruses persistence is critical for full elucidation of the mechanisms surrounding the interplay between viral persistence and host responses. Wildlife species that serve as natural host reservoirs are vital to the development of these models. To be clear, murine models are not what is being inferred here since these models have failed to answer important questions surrounding virus–host ecology. Therefore, future research should unite studies that encompass the molecular virology of vector-borne flaviviruses, molecular cell biology, accurate animal models, and studies involving virus–host interactions.

Surveillance models incorporating several variables, including ecological and climatic variables, as well as traits shared by vector-borne flaviviruses, have become a powerful tool in evaluating the potential function and role of wild-type hosts [187]. These models can be constructed to predict new host species with similar characteristics to known hosts of a particular flavivirus pathogen. Vector distribution information coupled with models for flavivirus host distribution permits the estimation of global susceptibility to flaviviruses, providing potential disease targets that are critical for disease surveillance in animals and humans. Of note, when obtaining host and flavivirus data for surveillance models, the exclusion of experimental infections of animals in laboratory settings should be performed to collect data only pertaining to hosts of vector-borne flaviviruses that can be infected through natural infection. When validating surveillance model results via laboratory testing, it is advised that for all flaviviruses studied, positive serological testing in a host is generally not considered conclusive (save for PRNT). This is due to inaccurate results resulting from potential cross-reactivity with other flaviviruses and inferior specificity. Therefore, the detection of a flavivirus by plaque reduction neutralization tests (PRNT) or PCR-based assays are considered to be more definitive in confirming surveillance model results [266].

Regarding all species studied (whether mammal, birds, reptiles, etc.), quite a few parameters need to be considered to properly construct a surveillance model predicting the distribution of potential hosts of vector -borne flaviviruses. Pertinent information, such as ecological traits, life history, and physiological data, is acquired from multiple databases. Moreover, additional information, such as population temporal trend data, taxonomy, conservation status, and habitat usage, are essential, all of which can be obtained from the International Union for Conservation of Nature (IUCN) [267]. Additional parameters to include in the model algorithm comprise metabolic rate, body temperature and body mass, and foraging characteristics (e.g., such as diet, foraging strata, and foraging time), which may be downloaded from databases BirdLife International and IUCN12 [267,268,269]. Global distribution data sets are also collected, which indicate the bearing of other factors, such as bioclimatic variables, mammalian and avian biodiversity, and anthropogenic variables, all of which are obtained from multiple sources [270,271,272]. Vector-host interactions, tick and mosquito biting preferences, and vector competence are all essential factors in maintaining virus circulation in a sylvatic environment. Due to the lack of availability of these data, a variable may be needed that describes the spatial distribution of vectors within the geographic range of a given host.

## 6. Conclusions

Persistent vector-borne infections in wildlife species serve as a reservoir for transmission to human hosts. For these infections, no FDA-approved therapy exists, leaving only the immune response to eliminate or contain the viral spread. However, ongoing viral activity in the absence of inhibitory immunity triggers outbreaks and even pandemics that lead to pathology in both reservoirs and downstream hosts via transmission by arthropod vectors, such as ticks and mosquitoes. Viral mechanisms contributing to persistence appear to include immune evasion strategies, through inhibition of interferon-mediated JAK/STAT pathway signaling and production of defective inhibitory virus particles, and through the activity of flavivirus non-structural proteins expressed during virus replication. Understanding the pathophysiology of these viral infections and the cellular mechanisms by which they establish persistence is key to the development of therapeutic approaches that can eliminate these persistent infections without immune suppression. Furthermore, it is important to consider that chronic, persistent infections of wildlife species could contribute to the re-emergence of pathogens in human host populations, with the current SARS-CoV-2 pandemic and the speculation of the pathogen’s origin in bat populations serving as a current example of the significance of this [273], and a reminder of why the continual study of viral persistence, especially flaviviruses, in wildlife populations is so critical.

## Figures and Tables

**Table 1:** Functions of flaviviral non-structural proteins implicated in viral persistence. Flavivirus non-structural proteins are summarized as they appear in the text. ** Where a function is not indicated for tick-borne flaviviruses (TBFV)s, a similar function is presumed for both tick and mosquito-borne flaviviruses since, in many cases, no detailed research endeavors have been carried out on TBFV.

Flavivirus Non-Structural Proteins	Mosquito-Borne Flavivirus	Tick-Borne Flavivirus **
NS1	NS1-mediated immune evasion leads to the activation of human complement by the specific inhibition of the classical and lectin pathways of complement activation through a direct interaction with complement components C4 and C1s [88]. For example, DENV, WNV, and YFV NS1 proteins have been shown to confine C4b expression and C3 convertase activity by increasing the digestion of C4 through the recruitment of the complement-specific protease C1s [88,89,90]. This effect might explain some of the clinical manifestations of dengue hemorrhagic fever and dengue shock syndrome. Each subunit forms a homodimer located in the ER lumen and co-localizes with viral dsRNA. Secreted and cell surface-associated NS1 is immunogenic and induces an antibody response that serves as a marker of flavivirus infection [91].	Degradation of TBEV-NS1 proteins also restricts TBEV replication [92]. Tick-borne encephalitis virus Far Eastern subtype (Sofjin virus) is associated with a 39-kDa truncated form of NS1 with acutely and persistently infected cells [93].
NS2A	Mutations within a pseudo-knot of NS2A RNA that is characteristic of the JEV subgroup abolish NS2A interaction with NS1, suggesting a role in viral neuroinvasiveness and attenuation in mice [94], and also suggests a potential link between neuroinvasiveness and flavivirus persistence in wildlife species.	
NS2B	NS2B is a hydrophobic protein that behaves as a cofactor for NS3. Together, they form a serine protease complex essential for processing the flavivirus polyprotein [95].	
NS3	NS3 protein is a critical member of the Replication Complex (RC) and is activated with NS5 to bind the genomic RNA prior to replication [96,97]. Mosquito-borne flavivirus NS3 can also potentially induce [98,99,100,101] INFα/β signaling [102]. For example, studies on neurovirulence associated with DENV-1 NS3 have shown that mutations in NS3 induce cell death in DENV-1-infected cells [103].	The protease domain of LGTV NS3 associates with caspase 8 and induces apoptosis [104].
NS4A	NS4A, in concert with NS3 and NS4B flavivirus proteins, is responsible for promoting the reorganization of host ER membranes, resulting in the development of virus-induced membranous spherules and vesicles enclosing the dsRNA and RC, potentially diminishing the exposure of actively replicating flavivirus RNA to innate immune sensor proteins, such as melanoma differentiation-associated gene 5 (MDA5) and retinoic acid-inducible gene I (RIG-I) [96,105,106], potentially aiding in the establishment of flavivirus persistence. The mature form of NS4A also induces PI3K-dependent autophagy signaling, leading to protection from antiviral-induced cell death [107].	
NS4B	The NS4B protein of DENV, JEV, and WNV, inhibits type I interferon (IFN-α/β) response through the inhibition of STAT1 phosphorylation [108,109,110]. The same is true for the WNV strain, NY99, which disrupts the host immune responses by blocking IFN-α/β/λ pathways through the disruption of the phosphoactivation of STAT1/STAT2, which is essential for nuclear translocation [108].	
NS5	The largest and most conserved among the vector-borne flavivirus proteins. NS5 primarily functions as the RNA-dependent RNA polymerase (RdRp) [97]. For WNV, NS5 is involved in different cellular pathways and has a crucial role in the escape from the IFN-α/β signaling pathway, typically through inhibiting phosphorylation of protein members of this pathway. DENV NS5-mediated inhibition of host TYK2 and STAT2 phosphorylation thwarts JAK-STAT signaling pathway activation [111].	Supplemental to functions described for the viral RdRp, NS5 described for the mosquito-borne viruses, and the NS5 of tick-borne flaviviruses were the first to be shown to disrupt innate immune signaling. For example, the suppression of critical host responses is shown through LGTV NS5 interactions with IFNAR2 and IFNGR2 (IFN receptor subunits) and antagonizes IFN-dependent responses via JAK-STAT signal transduction suppression [112,113]. The TBEV NS5 interacts with TRIM79-α, an IFN-inducible protein, leading to the inhibition of TRIM79-mediated degradation of TBEV-encoded proteins [86]. The ability of host cells to suppress tick-borne flaviviruses is compromised by the interaction of TBEV NS5 with a cellular scaffold protein, named Scribble, which blocks STAT1 phosphorylation and disrupts JAK-STAT-mediated signaling [114].

## Data Availability

Not applicable.
